# The emerging role of regulated cell death in ischemia and reperfusion-induced acute kidney injury: current evidence and future perspectives

**DOI:** 10.1038/s41420-024-01979-4

**Published:** 2024-05-04

**Authors:** Chenning Li, Ying Yu, Shuainan Zhu, Yan Hu, Xiaomin Ling, Liying Xu, Hao Zhang, Kefang Guo

**Affiliations:** 1 Department of Anesthesiology, Zhongshan Hospital, Shanghai, China; 2 Shanghai Key Laboratory of Perioperative Stress and Protection, Shanghai, China

**Keywords:** Apoptosis, Necroptosis

## Abstract

Renal ischemia‒reperfusion injury (IRI) is one of the main causes of acute kidney injury (AKI), which is a potentially life-threatening condition with a high mortality rate. IRI is a complex process involving multiple underlying mechanisms and pathways of cell injury and dysfunction. Additionally, various types of cell death have been linked to IRI, including necroptosis, apoptosis, pyroptosis, and ferroptosis. These processes operate differently and to varying degrees in different patients, but each plays a role in the various pathological conditions of AKI. Advances in understanding the underlying pathophysiology will lead to the development of new therapeutic approaches that hold promise for improving outcomes for patients with AKI. This review provides an overview of the recent research on the molecular mechanisms and pathways underlying IRI-AKI, with a focus on regulated cell death (RCD) forms such as necroptosis, pyroptosis, and ferroptosis. Overall, targeting RCD shows promise as a potential approach to treating IRI-AKI.

## Facts

- Ischemia/reperfusion-induced damage in proximal tubular epithelial cells (TECs) is considered the main cause of AKI.
- Various types of RCD have been linked to IRI, including necroptosis, apoptosis, pyroptosis, and ferroptosis.
- RCD-targeted therapies have shown effectiveness in curing IRI-AKI.

## Open questions

- What kind of RCD is most closely associated with IRI-AKI?
- Are there any other mechanisms through which RCD affects IRI-AKI? Do different types of RCD vary in their impact on IRI-AKI?
- When it comes to translating RCD-targeted therapies from experimental mouse models to clinical trials, what are the key considerations and challenges that need to be addressed? How can the findings and insights gained from mouse studies be effectively applied in the context of human patients undergoing clinical trials for IRI-AKI?

## Introduction

Acute kidney injury (AKI) is a complex syndrome that encompasses a range of conditions. It is characterized by a sudden decrease in kidney function that occurs within a period of 7 days, including an increase in serum creatinine levels and a reduction in urinary output [1]. AKI is a significant complication in approximately 15% of hospitalized patients [2]. AKI is even more common among patients in the intensive care unit (ICU) [3], with a prevalence of over 50%. Risk factors for AKI typically center around sepsis, major surgeries (including cardiac surgery) and drug toxicity [4]. During hospitalizations, certain factors are often present that can lead to generalized or localized ischemia. Ischemia/reperfusion is a pathological condition in which an organ experiences a temporary restriction of blood supply, followed by the restoration of perfusion and reoxygenation. However, this restoration often exacerbates tissue injury and triggers a profound inflammatory response [5]. Ischemia/reperfusion injury-related acute kidney injury (IRI-AKI) occurs when there is a mismatch between local tissue oxygen supply and demand. This results in sustained inadequate oxygen delivery, the accumulation of metabolites and ATP depletion, which can lead to ischemic acute tubular necrosis (ATN) and epithelial and endothelial injury if adequate renal perfusion is not restored. Ultimately, this causes activation of cell death programs and inflammatory processes, leading to renal dysfunction [6, 7]. Previous studies have suggested that tubular cell death by necrosis and apoptosis is the major mechanism associated with renal IRI [8]. However, recent research has challenged traditional views of cell death by identifying new pathways in which cells die in a regulated manner, including pyroptosis [9], necroptosis, and ferroptosis [10]. In this review, we summarize the current status of research on the molecular mechanisms and pathways of AKI induced by IRI. Advances in understanding the underlying pathophysiology will lead to the development of new therapeutic approaches that hold promise for improving outcomes for patients with AKI.

## Cell injury and dysfunction in IRI-AKI

### Epithelial cell injury

When blood flow to the kidneys decreases, the ATP levels in epithelial cells can drop, which may cause cellular injury or death. Although any part of the nephron can be affected, proximal tubular epithelial cells (TECs) are particularly vulnerable because of their high metabolic rate and limited ability to produce energy without oxygen. Ischemia-induced damage in proximal TECs is considered the main cause of AKI. Following injury, blood flow decreases, and congestion occurs in the outer stripe of the S3 segment, which can persist and contribute to ongoing ischemia. Ischemic cell injury causes the loss of the apical brush border of proximal TECs, exposing areas of the denuded tubular basement membrane, which leads to proximal tubular dilatation and the formation of distal tubule casts. Additionally, changes in the actin cytoskeleton and its dysfunction during ischemia alter cell polarity and function. Rapid depletion of ATP disrupts apical F-actin and tight junctions, resulting in increased permeability and back-leakage of the glomerular filtrate. Epithelial cells are also separated from the extracellular matrix due to integrin disruption, causing β-integrins to be relocated and viable cells to detach from the tubular basement membrane. The exfoliated cells then form cellular casts within the tubular lumen [6, 8]. Experiments have shown that mice with IRI-AKI exhibit distension, loss of cristae, and fragmentation of the mitochondria in transmission electron microscopy images. Additionally, there is higher expression of a marker of apoptosis (cleaved caspase 3) [11]. The mitochondrial dysfunction of TECs might be related to excessive formation of ROS and kidney damage in mouse models of IRI-AKI.

**Fig. 1:**
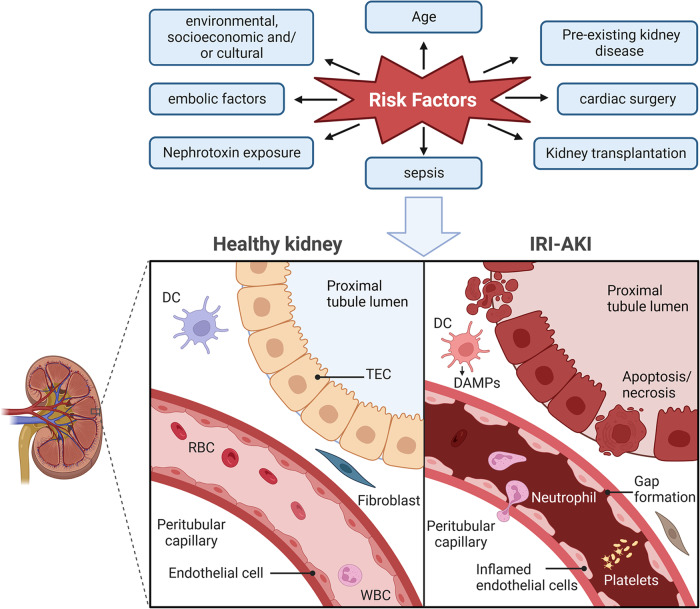
Human kidney biopsy with IRI-AKI (cortical area). Ischemia is a condition in which the kidneys do not receive enough blood supply. Reperfusion is the restoration of blood flow after an ischemic event. Both of these processes can damage kidney cells and impair kidney function, resulting in AKI. This can cause expansion of the interstitium, microvascular plugging, dilated tubules, and a patchy nature of injury, ultimately leading to various types of RCD. Several risk factors can contribute to the development of IRI-AKI. AKI acute kidney injury, IRI ischemia-reperfusion injury, RCD regulated cell death, TEC tubular epithelial cell, DC dendritic cells, RBC red blood cell, WBC white blood cell, DAMPs damage-associated molecular patterns.

TECs play both victim and perpetrator roles in IRI-AKI. A study found that TECs experience complex molecular and cellular events that are particularly relevant to IRI, including oxidative damage and activation of the innate immune system through Toll-like receptors (TLRs), sphingosine-1-phosphate (S1P) receptors, and hypoxia-inducible factors (HIFs). This complexity provides various physiological advantages, such as molecular messaging, cellular specificity, and response amplification. Additionally, proinflammatory cascades triggered by TECs promote the recruitment of leukocytes and dilation of the vasculature, affecting both the injured TECs and their surrounding microenvironment [12] (Fig. 1).

### Endothelial dysfunction

Endothelial cells play a role in regulating vascular tone, blood flow, coagulation, inflammation, and permeability. Ischemia can affect the renal endothelium, resulting in microvascular dysregulation and further injury. This injury can manifest as vascular congestion, edema, diminished blood flow, and inflammation. Interpreting total kidney blood flow after injury is challenging due to the complexity of the kidney’s vascular beds [8]. In postischemic kidney arterioles, vasoconstriction is amplified due to reduced production of vasodilatory substances by damaged endothelial cells and is augmented by vasoactive cytokines, causing them to constrict more than normal kidney vessels. Endothelial cells also contribute to IRI-AKI pathology by enhancing endothelium-leukocyte interactions, activating leukocytes, obstructing capillaries and postcapillary venules, and increasing microvascular permeability. In cases of renal hypoperfusion, epithelial cells may be damaged or killed via necrosis or apoptosis. In response to hypoxia, the functioning of adjacent injured or deceased TECs can be affected, leading to alterations in prostaglandin synthesis, the generation of reactive oxygen species, or the activation of inflammatory pathways. As a result, this can initiate inflammatory processes and injure the endothelium [7, 13]. Additionally, the number of microvessels in the inner stripe of the outer medulla declines after IRI, potentially leading to chronic hypoxia, increased tubular injury, and fibrosis [6].

Recent research shows that small membrane particles called extracellular vesicles (EVs) derived from endothelial colony-forming cells (ECFCs) may have the potential to treat AKI. EV treatment has advantages over stem or progenitor cells because it circumvents cell rejection, undesirable cells, and neoplasm formation [14]. Furthermore, molecular manipulation of EVs with exogenous factors and specific surface molecules can be used for renal endothelial cell therapy, which can improve the prognosis of IRI-AKI [15].

## Programmed cell death in IRI-AKI

**Fig. 2:**
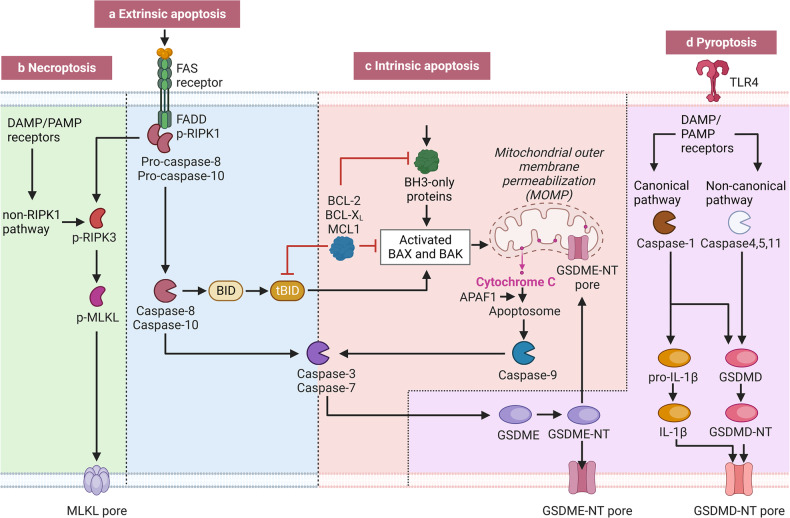
The interplay of molecular mechanisms in apoptosis, necroptosis, and pyroptosis. Both necroptosis and pyroptosis interact with apoptosis through the caspase family of proteins. **a** In the extrinsic apoptosis pathway, death receptors TNFR activate caspase-8, which in turn activates caspases and may also progress BID to tBID, activating the mitochondrial apoptotic pathway. Inhibitor of apoptosis (IAP) proteins activate NF-κB to increase the transcription of antiapoptotic proteins. **b** Necroptosis is programmed and regulated by RIPK1 and RIPK3, which form a necrosome in the presence of various stimuli and inhibition of caspase-8 and cIAPs. Phosphorylated MLKL induced by the necrosome oligomerizes to form pores that disrupt the plasma membrane, allowing the release of cell contents. **c** In the intrinsic apoptosis pathway, the imbalance between BCL-2 proteins and BH3-only proteins leads to mitochondrial outer membrane permeabilization. This causes the release of proapoptotic mitochondrial proteins, including cytochrome c, and the formation of the apoptosome (APAF1 and procaspase-9). Activation of caspase-9 then triggers the activation of executioner caspases (such as caspase-3 and caspase-7) that dismantle cell structures. **d** Pyroptosis is a process in which gasdermins are enzymatically processed into amino-terminal (NT) fragments. These fragments then assemble to form pores in the cell membrane. Danger signals, such as DAMPs, activate the inflammasome, which in turn triggers caspase-1 activation. Caspase-1 is involved in the maturation of IL-18 and IL-1β, and it also cleaves GSDMD to generate N-terminal fragments that create pores in the cell membrane. GSDMD can also be cleaved by caspases-4, 5, and 11 through a noncanonical pathway. Furthermore, gasdermins can cause mitochondrial permeabilization. There are also connections between pyroptosis and apoptosis, with caspase-3 cleaving gasdermin E (GSDME) and caspase-8 cleaving GSDMD.

Cell death, specifically the death of TECs, plays a significant role in the pathophysiology of IRI-AKI. Historically, cell death classifications were based on the morphological and structural details of individual tissues and cells. These physical characteristics have been used to classify cell death into three main types: apoptosis, autophagy, and necrosis [16]. The Nomenclature Committee on Cell Death (NCCD) has updated the classification system for cell death based on functional aspects: accidental cell death (ACD) is an uncontrolled biological process, whereas regulated cell death (RCD) involves tightly structured signaling cascades and molecularly defined effector mechanisms [17]. In addition to apoptosis, newly discovered forms of cell death, such as necroptosis, pyroptosis, and ferroptosis, are all examples of RCD. Recent studies suggest that different pathways for programmed cell death interact with each other (Fig. 2). The activation of these lytic cell death pathways leads to the release of inflammatory signals into the surrounding environment, which may prompt neighboring cells to die through different cell death modalities [18]. Further studies are needed to determine which cells in the kidney are most susceptible to injury and how the various modalities of cell death interact to either cause or prevent IRI-AKI. Novel therapeutic approaches can improve outcomes in patients with AKI by targeting specific cell death pathways.

Apoptosis is the most well-established form of cell death that occurs in IRI-AKI. The caspase family of proteases is responsible for initiating and executing apoptosis. Both intrinsic and extrinsic apoptotic pathways are activated in AKI. Intrinsic apoptosis is marked by mitochondrial outer membrane permeabilization (MOMP), while extrinsic apoptosis is initiated by perturbations in the extracellular microenvironment detected by plasma membrane receptors and propagated by caspase-8. Both pathways activate executioner caspases, mainly caspase-3 [16]. Effector caspases, such as caspase-3, -6, and -7, cleave numerous cellular proteins, which results in the classic apoptotic phenotype. Studies have shown that inhibiting caspase activity can be protective against injury [8]. The balance between cell survival and intrinsic apoptotic cell death is determined by the relative concentrations of proapoptotic proteins (Bax, Bad, and Bid) and antiapoptotic proteins (Bcl2 and Bclxl) of the Bcl-2 protein family. Overexpression of proapoptotic proteins or underexpression of antiapoptotic proteins can create pores in the outer mitochondrial membrane and possibly other intracellular membranes [16]. Other proteins involved in the apoptotic pathway include proximal-acting nuclear factors κB and p53. Kinases interact with signals from growth factors to mediate cellular responses involved in apoptosis, survival, and repair [8]. Preventing apoptosis of endothelial cells and TECs may be a potential therapeutic strategy for preventing AKI to CKD progression [19, 20].

Previous studies on kidney and cell death have focused on apoptosis as a cause of AKI. Experimental evidence supports that proximal tubule epithelial cells are highly susceptible to apoptosis and that apoptosis plays a pathogenic role in AKI [21]. However, early studies may not have clearly differentiated between apoptosis and regulated necrosis. These two processes share some of the same molecules and pathways. As a result, treatments targeting only apoptosis have been shown to be only partially effective [21].

### Pyroptosis in IRI-AKI

Pyroptosis is an inflammatory form of regulated cell death characterized by the activation of proinflammatory caspases and the formation of plasma membrane pores, which eventually leads to cell lysis [16, 22]. Previous studies have shown that pyroptosis mediates a variety of downstream effects by activating caspase-1 through the canonical pathway or caspase-4/5/11 through the noncanonical pathway [9]. In both pathways, gasdermin D (GSDMD) is the key effector of pyroptosis initiation. There is a growing body of evidence suggesting that pyroptosis may play a role in IRI. However, the role of pyroptosis and gasdermins in AKI has not been well studied and is not completely understood [23]. In 2014, in one of the earliest studies, Yang et al. discovered a significantly increased presence of pyroptosis-related proteins, such as caspase-1, caspase-11, and IL-1, following renal IRI. This study also revealed that CHOP/caspase-11, triggered by overactivated endoplasmic reticulum (ER) stress, may be an essential pathway involved in pyroptosis of renal TECs [24].

In the canonical pathway, activated inflammasome sensors (NLRP1, NLRP3, NLRC4, and AIM2) form the inflammasome complex with the adapter apoptosis-associated speck-like protein containing a CARD (ASC) for caspase 1 activation. Caspase 1 cleaves pro-IL-1β, pro-IL-18, and GSDMD, releasing the amino-terminal domain of GSDMD (GSDMD-NT) that forms pores in the plasma membrane. Pore formation by GSDMD mediates pyroptosis, causing the release of cellular contents, mature IL-1β and IL-18, and lytic cell death [23].

In contrast, the noncanonical pathway directly activates caspase-4/5/11 through oligomerization by binding with intracellular bacterial lipopolysaccharide (LPS), which cleaves GSDMD. A study showed that disulfiram improved renal impairment after IR by blocking the upregulation of noncanonical pyroptosis pathway proteins, suggesting that it might reduce pyroptosis by antagonizing TLR4 and inhibiting the caspase-11/GSDMD pathway [25].

Although all of the studies mentioned above indicate that GSDMD is the key contributor to renal IRI, a recent study provided the first evidence that GSDMD-positive cells may function as a suppressor of AKI rather than being primarily involved in renal TEC death. Specifically, these cells constrain necroptotic cell death in the tubules through a previously unknown noncell autonomous crosstalk with the necroptosis machinery [26]. Overall, most experimental studies investigating pyroptosis in AKI have been conducted using mouse models, and the results of these studies are quite inconsistent. Therefore, further research is needed to determine the role of this form of cell death in human AKI and its association with IRI.

### Necroptosis in IRI-AKI

Necroptosis is a type of regulated cell death that leads to cell lysis and inflammation in nearby tissues, which is typically characterized by the morphological features of necrosis. This pathway is often associated with kidney disease and is defined at the molecular level by the involvement of three proteins: receptor-interacting serine/threonine protein kinase 3 (RIPK3), RIPK1, and the executioner pseudokinase mixed-lineage kinase domain-like protein (MLKL) [18]. The necroptosis pathway induced by TNF-α has been extensively researched [27]. Although necroptosis is commonly perceived as a highly proinflammatory mode of cell death due to its rapid lysis of cells and liberation of DAMPs, some researchers suggest that it might have anti-inflammatory effects in certain contexts. Specifically, it may reduce excessive TNF- or TLR-induced inflammatory cytokine production [28]. IRI occurs when the blood and oxygen supply to the kidney is temporarily restricted, leading to depleted ATP and accumulated metabolites. Upon restoration of blood flow, necroptosis occurs, causing acute tubular necrosis and endothelial damage, which ultimately leads to inflammation [18].

There is a body of evidence indicating that a lack of RIPK3 and MLKL expression can reduce kidney damage due to IRI, and the necroinflammation driven by RIPK3/MLKL-dependent necroptosis may lead to the progression of IRI to CKD [29]. Additionally, the accumulation of pMLKL was found in the necrotic tubules of human patients with AKI [30]. Furthermore, Feng et al. discovered that in response to renal IRI, RIPK3 translocates into mitochondria and interacts with an inner mitochondrial protein (mitofilin). This interaction promotes the release of mitochondrial DNA (mtDNA) into the cytosol, which activates the cGAS/STING pathway, leading to increased nuclear transcription of proinflammatory markers, further exacerbating renal IRI [31]. However, the interplay between RIPK3 and MLKL remains unclear and requires further study. The extent to which the effects of RIPK3 deficiency, MLKL deficiency and the combined deficiency on IRI differ is unclear [32, 33]. RIPK3 may worsen kidney injury even without the involvement of MLKL and necroptosis [33].

In addition, either apoptosis or necroptosis can be initiated by receptors such as TNFR and Fas, depending on the intracellular state of the target cell. Based on the significant amount of necrotic tubular cells, Linkermann et al. concluded that renal IRI primarily occurs through necroptosis, rather than apoptosis, and can be attenuated by inhibiting RIP1-mediated necroptosis by necrostatin-1 (nec-1) [34, 35]. However, subsequent studies have indicated that nec-1 could potentially inhibit ferroptosis and that ferroptosis might be more significant than necroptosis in renal IRI [18, 36]. In 2014, Linkermann et al. suggested that renal tubules are not sensitized to necroptosis by loss of FADD or caspase-8, and the RIPK1 inhibitor nec-1 does not protect freshly isolated tubules from hypoxic injury, while ferroptosis directly causes synchronized necrosis of renal tubules in models of IRI-AKI [37]. Another point of view is that necroptosis and ferroptosis function independently in many pathologies that involve cell death, but that they are alternative pathways in murine renal IRI. This interconnection reflects that sensitization to one pathway can result in resistance to the other pathway, while compensation from one pathway can occur when the other is compromised [38]. In conclusion, although most experimental studies investigating necroptosis in IRI-AKI have been conducted using mouse models, our understanding of the dynamics of necroptosis in human IRI-AKI remains inadequate. Additionally, further studies are needed to explore the interplay between necroptosis and other types of cell death.

### Ferroptosis in IRI-AKI

**Fig. 3:**
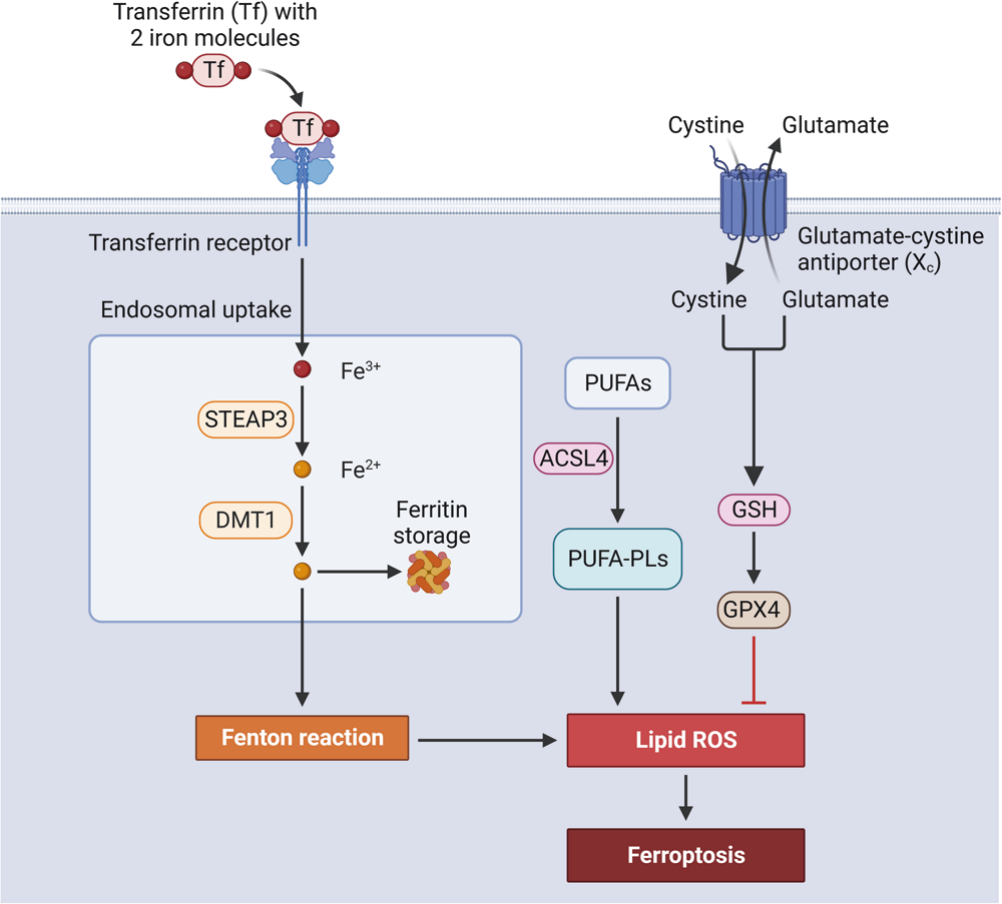
Main mechanisms of ferroptosis. Ferroptosis is a process characterized by the accumulation of lipid ROS. To prevent this accumulation, sufficient levels of GSH are maintained through the uptake of cystine via the system xc−cystine/glutamate antiporter. The antioxidant function of GPX4 depends on GSH. Another antioxidant system involves FSP1, which helps maintain vitamin K and CoQ10 in a reduced state. The main outcome of ferroptosis is the peroxidation of membrane phospholipids, resulting in membrane rupture. ROS reactive oxygen species, GPX4 glutathione peroxidase 4, GSH glutathione, CoQ10 coenzyme Q10.

Ferroptosis was first proposed by Dixon et al. in [39]. It is an iron-dependent form of regulated necrotic cell death characterized by the accumulation of lipid ROS, which differs from other forms of cell death in terms of its morphology, biochemistry, and genetics [39]. The most important feature underlying the distinction is lipid peroxidation in the intracellular microenvironment, which relies on the generation of ROS and the availability of iron [40]. This process leads to membrane destruction but can be controlled by glutathione peroxidase 4 (GPX4) [16]. The regulatory pathways of ferroptosis can be divided into three main groups: the GSH/GPX4 pathway, iron metabolism-related pathways, and lipid metabolism-related pathways [41] (Fig. 3).

As a crucial regulator of ferroptosis, many studies have investigated the role of GPX4 in ferroptosis associated with IRI-AKI. If GPX4 activity is inhibited, lipid peroxides can accumulate. This buildup is a crucial factor in the occurrence of ferroptosis, and GPX4 is the primary regulator of this process [41]. In 2014, genetic evidence showed that the knockout of GPX4 leads to ferroptosis, and by using inducible GPX4^−/−^ mice, it was found that the glutathione/GPX4 axis plays an essential role in preventing lipid oxidation-induced AKI [42]. In 2019, Huang et al. first found that I/R-induced kidney ferroptosis was mediated by augmenter of liver regeneration (ALR) via the GSH/GPX4 axis [43]. A recent study suggested that GPX4 is a substrate of tripartite motif containing 21 (TRIM21) and can be degraded by TRIM21-mediated ubiquitination, suggesting that inhibiting TRIM21 attenuates ferroptosis. Loss of TRIM21 negatively regulates ferroptosis by upregulating GPX4, alleviates IRI-AKI, and improves renal function [44]. Another member of the TRIM family, TRIM27, is involved in ubiquitin-specific peptidase 7 (USP7)-related ferroptosis. Inhibition of USP7 attenuated IRI-AKI by inhibiting ferroptosis through decreasing the ubiquitination of TRIM27-mediated TANK-binding kinase 1 (TBK1) and promoting DNA methyltransferase 1 (DNMT1)-mediated methylation of FMRP translational regulator 1 (FMR1) [45].

Another essential component for executing ferroptosis is acyl-CoA synthetase long-chain family member 4 (ACSL4), as it influences the necessary lipid composition in ferroptosis [46]. In 2020, Zhao et al. first discovered ferroptosis in TECs of patients with clinical kidney disease, confirming the correlation between ACSL4 and kidney function decline. They also identified a potential ferroptosis inhibitor, XJB-5-131, which showed promising results by alleviating kidney injury, promoting TEC proliferation and repair, decreasing kidney inflammation, and inhibiting lipid peroxidation accumulation in the I/R model [47]. In 2023, the same team showed that cytosolic high-mobility group box 1 (HMGB1) can cause ferroptosis by binding with ACSL4 after IRI. This study showed that inhibiting HMGB1 nucleocytoplasmic translocation pharmacologically and deleting HMGB1 in TECs in mice were effective in preventing IRI-AKI, tubular ferroptosis, and inflammation compared to control groups [48]. Previous studies have demonstrated the significance of ferroptosis in AKI. Targeting specific genes or proteins involved in ferroptosis-related pathways could offer potential therapeutic targets for treating IRI-AKI.

### New therapeutic targets: pyroptosis, necroptosis and ferroptosis

Renal IRI is a major contributor to AKI, for which an effective treatment has yet to be identified. We have compiled a list of clinically viable targeted drugs (Table 1) for RCD. Gasdermins and pyroptosis significantly impact tissue homeostasis, inflammation, and disease development. Approaches to attenuate renal IRI include inhibiting NLRP3 inflammasome activation and activating the Nrf2/NLRP3 signaling pathway. Other pathways, such as cholecalciferol pretreatment and Tisp40 overexpression, affect pyroptosis in IRI-AKI. Several novel inhibitors of necroptosis, such as Nec-1f, have shown promise in treating AKI in experimental models of renal IRI. The E-prostanoid 3 receptor (EP3) is another potential target for disrupting necroinflammation and improving ischemic AKI. Therapeutic strategies targeting Ferroptosis involve controlling lipid peroxidation, manipulating thiol metabolism, and regulating specific miRNAs and heme oxygenase-1. It is evident from the tables that current studies on drug therapy are being conducted in animal models. However, certain medications and small molecule inhibitors targeting regulated cell death pathways have shown promise in experimental models of renal IRI.

**Table 1:** Agents targeting RCD that can be used for IRI-AKI.

Drug name	Daily use	RCD	Subject	I/R model	Protect effect	Reference
Hydrogen sulfide (H2S)	No (an endogenous gas transporter)	Pyroptosis	Mouse, HK-2 cells	Bilateral renal pedicle clipping for 30 min	Inhibit the activation of the inflammasome NLRP3	[50]
β-hydroxybutyrate (β-OHB)	No (ketone bodies)	Pyroptosis	Mouse, HK-2 cells	Two weeks before the study, the right kidney was removed. After a 2-week recovery period, the left renal artery and vein were occluded for 45 min.	Inhibit the activation of the inflammasome NLRP4, epigenetic effect on FOXO3 expression	[51]
Activated protein C	No	Pyroptosis	Mouse	Bilateral renal pedicle clipping for 30 min,followed by 24 h of reperfusion	Inhibit the activation of the inflammasome NLRP5	[52]
Transient receptor potential-6 (TRPC6)	No	Pyroptosis	Mouse, HK-2 cells	Bilateral renal pedicle clipping for 30 min,followed by 24 h of reperfusion	Inhibit the activation of the inflammasome NLRP6	[53]
Salvianolic acid B (SalB)	A water-soluble compound extracted from Salvia miltiorrhiza	Pyroptosis	Mouse, HK-2 cells	Left renal pedicle clipping for 45 min, including contralateral nephrectomy	Inhibit NLRP3 inflammatory vesicle activation and prevent burning-induced inflammation by decreasing intracellular ROS	[54]
Naringenin	Flavonoids	Pyroptosis	Mouse, HK-2 cells	The left renal artery was clamped 30 min after right-sided nephrectomy, followed by 24 h of reperfusion	Inhibit inflammation by inducing HO-1 expression	[56]
Necrostatin-1(Nec-1)	No	Necroptosis	Mouse, HT29 cells	Bilateral renal pedicle clipping for 30 min	Rip1 inhibitor	[35]
Nec-1f	No	Necroptosis	Mouse, primary murine renal tubules	Bilateral renal pedicle clipping for 30 min	Inhibit RIPK1 and weakly inhabit ferroptosis	[62]
XJB-5-131	No (has shown beneficial effects in degenerative diseases)	Ferroptosis	Mouse	Unilateral renal artery occluded for 30 min	Ferroptosis inhibitor	[48]
Dexmedetomidine (Dex)	α2-adrenergic receptor (α2-AR) agonist	Ferroptosis	Mouse, Human Embryonic Kidney (HEK) 293 T cells	Bilateral renal hilum clipping for 45 min and followed by 48 h reperfusion	Suppress ACSL4, mitigate tissue damage, inhibit ferroptosis, and downregulated inflammation response following renal IRI	[66]
USC-Exo	No (exosomes)	Ferroptosis	Mouse, HK-2 cells	Bilateral renal pedicle clipping for 45 min	Interact with SRSF1	[67]

### Therapeutic significance of pyroptosis in IRI-AKI

Although gasdermins and pyroptosis are not yet fully understood, they are thought to have a significant impact on tissue homeostasis, inflammation, and the development of various diseases. One approach is to prevent the assembly of caspase-1-activating inflammasomes. In response to various PAMPs and DAMPs, NLRP3 oligomerizes with the adapter protein ASC to form a high molecular weight complex that recruits and cleaves pro-caspase-1, activating it and driving the maturation of IL-1β and IL-18, GSDMD cleavage, and pyroptosis. Studies have found that there are several ways to ultimately attenuate renal IRI by inhibiting the activation of the NLRP3 inflammasome, such as using hydrogen sulfide (H2S) [49], β-hydroxybutyrate (β-OHB) treatment [50], inhibiting mTORC1 with activated protein C (aPC) [51], and promoting zinc ion influx in renal TECs with transient receptor potential-6 (TRPC6) [52]. The cleavage of GSDMD by another caspase, caspase-11, promotes pyroptosis and IL-18 release in PTCs, playing a significant role in AKI. Therapeutically targeting both steps of Nlrp3 inflammasome with aPC and parmodulin-2 or small compound Nlrp3 inhibitors appears promising [53]. The cleavage of GSDME by Caspase-3 is implicated in the formation of membrane pores, cell lysis, and the exacerbation of renal tubular injury and inflammation. Treatment with Z-VAD-FMK, a broad-spectrum caspase inhibitor, led to a notable decrease in the levels of GSDME-N. These observations suggest that partial inhibition of pro-pyroptotic factors could potentially ameliorate pyroptosis and associated tissue damage [54].

Moreover, nuclear factor erythroid 2 related factor 2 (Nrf2) is a significant regulator of redox balance that controls both proinflammatory and anti-inflammatory effects, but its detailed mechanism remains unknown. Nrf2 can inhibit NLRP3 inflammatory vesicle activation and prevent burn-induced inflammation by decreasing intracellular ROS. For example, salvianolic acid B (SalB) can inhibit caspase-1/GSDMD-mediated pyroptosis by activating the Nrf2/NLRP3 signaling pathway, resulting in alleviation of IRI in mice [55]. In addition, Nrf2 is known to have an inhibitory effect on inflammation by inducing HO-1 expression. Inhibiting PRMT5 [56] or administration of naringenin [57] can activate the Nrf2/HO-1 pathway, which may alleviate pyroptosis and ameliorate renal IRI in mouse models. Further studies are necessary to clarify the regulation of inflammatory vesicles and burn injury by Nrf2.

Other pathways are also involved in pyroptosis in IRI-AKI. It appears that cholecalciferol pretreatment may reduce GSDMD-mediated pyroptosis by decreasing ROS production and NF-κB activation [58]. Conversely, overexpression of Tisp40 increases TEC GSDMD-mediated pyroptosis and related protein expression via NF-κB signaling [59].

The study of gasdermins and pyroptosis in the kidney is a relatively new area of research. Therefore, it is premature to draw any firm conclusions about their roles in renal pathogenesis based on the literature. Currently there are no commercially available drugs that block the pyroptosis pathway to prevent AKI, but rather inhibitors of specific molecules. Further research is needed before gasdermins or pyroptosis can be considered potential treatment targets for human kidney disease. It is possible to start with existing drugs to discover their relationship with the pyroptosis in IRI-AKI. Viable treatment options have been reported in other causes of AKI, which might provide new directions for the research of IRI-AKI. Fibroblastic reticular cells (FRCs) derived exosomes (FRC-Exos) can improve kidney function in sepsis-induced acute kidney injury (S-AKI). FRC-Exos promote PINK1-dependent mitophagy and inhibit NLRP3 inflammasome activation in lipopolysaccharide (LPS)-stimulated primary kidney tubular cells (PKTCs) [60]. It is important to validate any observations made from animal models in humans before clinical trials can be conducted to test treatments targeting gasdermins, pyroptosis or their upstream regulators in patients with kidney disease.

### Therapeutic significance of necroptosis in IRI-AKI

Several novel inhibitors of necroptosis are effective in experimental models of renal IRI, making them promising targets for the treatment of AKI. Most of them inhibit necroptosis and ameliorate AKI by targeting RIPK1 and/or RIPK3 [61–63]. Tonnus et al. developed a combined small molecule inhibitor called Nec-1f, which can potently inhibit RIPK1 and mildly inhibit ferroptosis and has been shown to improve survival in models of IRI-AKI [64]. Some inhibitors of AKI act through an MLKL-dependent mechanism. For example, repulsive guidance molecule (RGM)-b has been shown to inhibit MLKL membrane association and necroptosis in proximal TECs in mouse models [65]. Another promising target is the E-prostanoid 3 receptor (EP3). Deficiency of this receptor on myeloid cells can ameliorate ischemic AKI by disrupting the autoamplification loop of necroinflammation [66].

The anticonvulsant Phenytoin, classified as a hydantoin, was identified as an RIPK1 inhibitor that shows promise in the inhibition of necroptosis, albeit with lower potency compared to other inhibitors [30]. A novel small molecule Hsp90 inhibitor, C-316-1, attenuates acute kidney injury by suppressing RIPK1-mediated inflammation and necroptosis. The compound disrupts the Hsp90–Cdc37 protein–protein interactions, leading to a decrease in RIPK1 and reduced necroptosis. Despite the lack of approved Hsp90 inhibitors on the market due to toxicity concerns, C-316-1 shows promise due to its lower toxicity and better water solubility [67]. Augmenter of liver regeneration (ALR), a multifunctional factor known for promoting hepatocyte proliferation, has been found to have antiapoptotic and anti-oxidative stress effects and shows promise as a protective agent against AKI. Exogenous injection of ALR has a protective effect against AKI, and overexpression of the ALR gene decreases mitochondrial damage [68].

While these medicines and small molecule inhibitors of necroptosis have demonstrated effectiveness in experimental models of renal IRI, our understanding of the dynamics of necroptosis in human IRI is limited and likely to hinder the clinical use of necroptosis inhibitors. Existing drugs are not yet ready for use, either because they are too toxic or because they are ineffective. Further exploration is warranted to fully understand its potential as an affordable and readily accessible treatment option, including the determination of in vivo concentrations in specific target organs. The potential correlation between the long-term side effects, such as gingival hyperplasia of phenytoin, should also be investigated. So, it is possible to start with what is already available and improve them.

### Therapeutic significance of ferroptosis in IRI-AKI

Ferroptosis is implicated in the pathogenesis of kidney disease and could be targeted for therapy. This process occurs in cells with a redox imbalance and leads to cell death if other protective mechanisms fail. The excessive accumulation of certain products of phospholipid peroxidation triggers this process. Since iron and thiol imbalance can induce phospholipid oxidation through multiple pathways, there is potential for developing various regulators [69].

Among these therapeutic strategies, the control of lipid peroxidation might represent the most attractive strategy. ASCL4 plays a crucial role in synthesizing phospholipids that contain arachidonic acid, which are particularly vulnerable to oxidation during ferroptosis. Therefore, targeting ASCL4 could be a promising strategy for developing anti-ferroptosis therapies.

Dexmedetomidine, an α2-adrenergic receptor (α2-AR) agonist, has been shown to have renal protective effects against ferroptosis-mediated renal IRI and the inflammatory response through the suppression of ACSL4 [70]. lncRNA TUG1 carried by human urine-derived stem cell (USC)-derived exosomes (USC-Exo) regulates ASCL4-mediated ferroptosis by interacting with SRSF1 and then protects against IRI-induced AKI [71]. In addition, Shi et al. indicated that miR-20a-5p negatively regulated ACSL4 by targeting the 3’ UTR of ACSL4 mRNA, thereby inhibiting ACSL4-dependent ferroptosis and alleviating kidney IRI [72].

Another approach to targeting lipid peroxidation is through the use of radical-trapping antioxidants such as ferrostatin-1 [73] or liproxstatin-1 [74]. As previously mentioned, necrostatin-1, an inhibitor of RIPK1 and a crucial player in necroptosis, also hinders ferroptosis [36].

Targeting thiol metabolism can be an effective strategy. Small molecule compounds that boost GSH levels and GPX4 activity have been shown to inhibit ferroptosis, while depriving cells of cysteine and inhibiting GPX4 can trigger ferroptosis. However, directly administering GSH is not currently a practical option owing to its limited bioavailability and inability to pass through the plasma membrane [69, 75]. Some studies have found that other miRNAs can play a regulatory role in ferroptosis in IRI-AKI. According to Ding et al., there was an upregulation of miR-182-5p and miR-378a-3p following IRI, resulting in the activation of ferroptosis in renal injury through the downregulation of GPX4 and SLC7A11 [76]. Moreover, heme oxygenase-1 (HMOX1) is significantly upregulated during the early stages of IRI in the kidneys. Inhibiting miR-3587 promotes upregulation of the HO-1 protein, which is encoded by HMOX1, and thus protects renal tissues from IR-induced ferroptosis [77].

Cyanidin-3-glucoside (C3G), an anthocyanin, can attenuate acute organ injury and modulate oxidation, which markedly ameliorated Era-induced ferroptosis, resulting in nearly equivalent effects as selective ferroptosis inhibitor Lip-1 [78]. In a similar fashion, Entacapone, a medication commonly prescribed for Parkinson’s disease, has exhibited notable efficacy in preventing renal IRI by inhibiting ferroptosis. It achieves this by boosting antioxidant defenses through modulation of the p62-KEAP1-NRF2 pathway and the upregulation of SLC7A11 expression. Consequently, this intervention effectively suppresses oxidative stress and ferroptosis [79]. Additionally, Legumain, a cysteine protease known for its role in promoting ferroptosis by interacting with the ferroptosis inhibitor GPX4, is being investigated as a potential therapeutic target and an early diagnostic marker for IRI-AKI [80].

The exact mechanism of ferroptosis in IRI-AKI remains to be determined experimentally. While ferroptosis-specific inhibitors, such as ferrostatin-1 (Fer-1) and liproxstatin-1 (Lip-1), have shown protective effects in scientific research, they are not yet ready for clinical use. Drug discovery is at a basic stage, and it is still very far from clinical application. Therefore, re-evaluation of marketed drugs is an important way to achieve breakthroughs rapidly. Further research should explore ways to regulate ferroptosis to improve AKI. These findings will provide evidence for clinical translation.

## Conclusion and perspectives

The process of IRI-AKI involves multiple mechanisms of cell injury, cell dysfunction and various types of cell death. This paper outlines the preliminary understanding of the mechanisms underlying its incidence, characteristics, and targeted signaling pathways. Although most studies targeting IRI-AKI-related signaling pathways have been conducted in mouse models or in vitro experiments, exploring the role played by IRI in AKI and using potential targets to regulate AKI could provide new perspectives and therapeutic strategies to improve outcomes for AKI patients.

While current experimental techniques have been unable to determine the most significant form of cell death in IRI-AKI or whether they interact with each other, studies using animal models have provided increasing amounts of evidence of a correlation between RCD and IRI-AKI. The exact mechanism of RCD in the development and progression of IRI-AKI has yet to be determined experimentally. To do so, more functional studies that involve specific molecular markers and targeted inhibitors are necessary. In recent years, a significant amount of work has been done to identify biomarkers of cell death in vivo.

A novel form of cell death called PANoptosis has been discovered, involving multiple programmed cell death pathways such as pyroptosis, apoptosis, and necroptosis. Crosstalk among these pathways reveals new insights into the roles of inflammasome sensors and cell death complexes in disease. Caspase-8, traditionally linked to apoptosis, also plays a role in regulating the NLRP3 inflammasome and activating pyroptotic molecules. Caspase-3, a key player in apoptosis, can trigger pyroptosis as well, leading to both lytic and non-lytic cell death [81]. This unique process, known as PANoptosis, involves the activation of various cell death mechanisms simultaneously, leading to rapid and irreversible loss of cell viability. Research is ongoing to investigate the underlying mechanisms and implications of PANoptosis, with a focus on the role of NLRP12 in initiating this form of cell death in conditions such as AKI and hemolytic disease [82]. Enhancing the understanding of PANoptosis in the context of IRI-AKI has the potential to offer invaluable insights into the intricate interplay of cell death pathways in this condition. With the complexities surrounding IRI-AKI pathogenesis, delving into how PANoptosis interacts with conventional cell death mechanisms like apoptosis and necrosis could unveil innovative therapeutic targets for alleviating kidney damage and enhancing patient outcomes. Through unraveling the role of PANoptosis in IRI-AKI, researchers may discover new avenues for developing targeted interventions that target the specific mechanisms underlying renal injury and promote tissue regeneration.

When translating RCD-targeted therapies from experimental mouse models to clinical trials for IRI-AKI, there are several key considerations and challenges that need to be addressed: relevance of mouse models, dosing and pharmacokinetics, safety and toxicity, biomarkers and endpoint selection, patient heterogeneity, and regulatory approval. To effectively apply the findings and insights gained from mouse studies in the context of human patients undergoing clinical trials for IRI-AKI, collaboration between basic scientists, clinicians, and regulatory authorities is key. Robust preclinical data, including mechanistic insights and efficacy in relevant animal models, should guide the design of clinical trials. Additionally, close monitoring of patient responses, careful interpretation of clinical data, and adaptive trial designs are important strategies to optimize the translation of RCD-targeted therapies for IRI-AKI from bench to bedside.

Accurately identifying the form of RCD is of utmost importance, as it can serve as a solid diagnostic foundation for IRI-AKI and other related illnesses. It is imperative to conduct further research on how to effectively regulate RCD to enhance treatment strategies for IRI-AKI. These discoveries will ultimately offer supporting evidence for clinical translation.
